# On Trees with Greatest *F*−Invariant Using Edge Swapping Operations

**DOI:** 10.1155/2022/8291974

**Published:** 2022-06-28

**Authors:** Wenhu Wang, Adnan Aslam, Muhammad Ahsan Binyamin, Salma Kanwal, Iqra Irshad

**Affiliations:** ^1^School of Software, Pingdingshan University, Pingdingshan, Henan 467000, China; ^2^International Joint Laboratory for Multidimensional Topology and Carcinogenic Characteristics Analysis of Atmospheric Particulate Matter PM2.5, Pingdingshan, Henan 467000, China; ^3^College of Computing and Information Technologies, National University, Manila PH1008, Philippines; ^4^University of Engineering and Technology, Lahore(RCET), Pakistan; ^5^Department of Mathematics, GC University Faisalabad, Pakistan; ^6^Department of Mathematics, Lahore College for Women University, Lahore, Pakistan

## Abstract

The *F*-index of a graph *Q* is defined as *F*(*Q*)=∑_*t*∈*V*(*Q*)_(*d*_*t*_)^3^. In this paper, we use edge swapping transformations to find the extremal value of the F-index among the class of trees with given order, pendent vertices, and diameter. We determine the trees with given order, pendent vertices, and diameter having the greatest *F*-index value. Also, the first five maximum values of *F* index among the class of trees with given diameter are determined.

## 1. Introduction

Mathematical chemistry is providing effective and time-saving methods for evaluating the properties of chemical compounds without having to go through tedious laboratory experimentations. Topological indices are function maps that identify key computational and topological aspects of a structure and evaluate chemical compound properties without using quantum mechanics as final production [1]. The total *π*-electron energy (*E*) [2] of a molecule was found to be related to its thermodynamic stability that depends on the structure of a molecule that is its topology. Relationship between (*E*) and topology of a molecule was determined by its graphical structure [3]. Comparison was made between the original vertex degree-based indices and lately defined edge degree-dependent indices (termed as reformulated Zagreb indices), while relating the two versions of indices, the relation existing between the graph and its line graph was utilized. Yang et.al. [4] brought into consideration to researchers the relation between the subtree number index and the Weiner index in the class of spiro chains and polyphenyl hexagonal chains.

In this paper, we consider only simple finite and connected graphs. In a graph *Q*, we denote its vertex set and edge set by *V*(*Q*) and *E*(*Q*), respectively. Let *d*_*Q*_(*p*) denotes the degree of a vertex *p*. The distance between two vertices *p*, *t* ∈ *V*(*Q*) is denoted by *d*(*p*, *t*) and is defined as the length of the shortest path joining them. For more undefined terminologies related to graph theory, we refer [5].The first topological index were proposed by Weiner [6] (namely, the Weiner index), while he was working on the boiling point of paraffin. The Weiner index is denoted by *W*(*Q*) and is defined as

(1) $$\begin{array}{c} W\left(Q\right)=\sum_{\left\{p,t\right\}\subseteq V\left(Q\right)}d_{Q}\left(p,t\right). \end{array}$$

Zagreb indices were introduced by Gutman et al. [2] that depend on degrees of nodes and are defined as

(2) $$\begin{array}{c} M_{1}\left(Q\right)=\sum_{p\in V\left(Q\right)}{\left(d_{p}\right)}^{2}\\ M_{2}\left(Q\right)=\sum_{pt\in E\left(Q\right)}d_{p}d_{t}. \end{array}$$

These terms were recognized to be a measure of the extent of branching of the carbon atom skeleton of the underlying molecule. Later, its additive version was brought into kind attention to researchers in [7], which as expected, revealed more hidden chemical properties of chemical compounds. This index is named as the general sum connectivity index, given as

(3) $$\begin{array}{c} \chi_{\alpha }\left(Q\right)=\sum_{pt\in E\left(Q\right)}{\left(d_{p}+d_{t}\right)}^{\alpha }. \end{array}$$

Furtula *et.al.* [8] in 2015 introduced the *F*-index, also referred as the forgotten topological index, which is defined as

(4) $$\begin{array}{c} F\left(Q\right)=\sum_{t\in V\left(Q\right)}{\left(d_{t}\right)}^{3}. \end{array}$$

This index is also a measure of branching and has same measure of predictability as that of the first Zagreb index. In case of the acentric factor and entropy, both *M*_1_(*Q*) and *F*(*Q*) have a correlation coefficient greater than 0.95 [8].

Ali et al. [9] put forward the survey of work done on the Randic index for certain values of *α*. Azari et al. [10] considered the forgotten topological index in detail and determined the bounds of this index in terms of other graphical parameters. They analyzed the relationship of this index with already exiting versions of Zagreb indices. Z. Che et al. [11] determined new bounds for the forgotten index in terms of graph irregularity, Zagreb indices, and many other existing graph invariants. Further they characterized the graphs attaining these bounds and proved that these newly attained bounds are sharper than the existing ones. Another version of the forgotten index namely the forgotten co-index was brought into attention by Ghalavand et al. [12]. The authors found bounds for this index and provided an ordering of graphs with respect to this index. Gutman et al. [13] provided a finite ascending sequence of the forgotten index for trees and moreover for graphs having some particular values of the cyclomatic number *γ*. Gutman et al. [14] proved two weighted inequalities of real nonnegative sequences and then used them to determine lower bounds of certain degree dependent indices.

The main motivation behind this work is the idea practiced in [15], in which authors introduced some edge swapping operations on graph structures and analyzed the behavior of generalized sum connectivity descriptor. The authors found the decreasing behavior of the descriptor and provided the least five values of this descriptor for trees. Further they also provided the trees that attain these least values. In this work, making use of certain graph transformations that involve the swapping of edges from one node to another and contraction of edges, we have observed the behavior of the *F*-index. This enabled us to determine the decreasing sequence of values of *F*-invariant and the corresponding trees attaining these values. Novelty of work lies behind the fact that solving a research problem that is not solved already is always a good addition to the existing literature. Thus, this problem of determining members in a certain family of graph with first, second up to fifth extremal values has become good source of attraction to researchers.

## 2. *F*-Invariant under Certain Transformations

In this section, we first observe the increasing or decreasing behavior of *F*-invariant under certain graph operations involving swapping of edges from one node to another. Our next results show that this descriptor exhibit increasing behavior.

(5) $$\begin{array}{c} \alpha_{1}-\text{transform.} \end{array}$$

**Figure 1:**
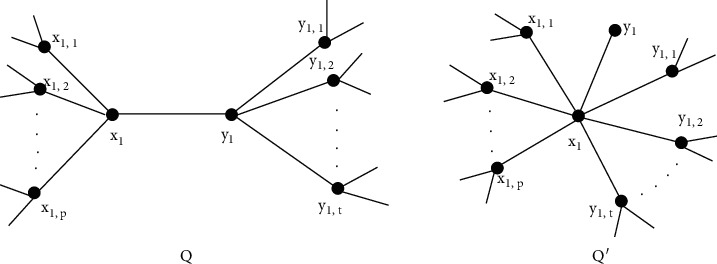
*α*_1_ − transform applied to *Q*.

Let *Q* be the connected tree and *x*_1_, *y*_1_ ∈ *V*(*Q*). For *p* ≥ 0, *t* ≥ 1, suppose *N*(*x*_1_)={*y*_1_, *x*_1,1_, *x*_1,2_,…, *x*_1,*p*_} and *N*(*y*_1_)={*x*_1_, *y*_1,1_, *y*_1,2_,…, *y*_1,*t*_}, where the vertices *x*_1_ and *y*_1_ have no common neighbors in *Q*. Let *α*_1_(*Q*) be the graph derived from *Q* by deleting edges *y*_1_*y*_1,1_, *y*_1_*y*_1,2_,…, *y*_1_*y*_1,*t*_ and attaching new edges *x*_1_*y*_1,1_, *x*_1_*y*_1,2_,…, *x*_1_*y*_1,*t*_. We say that *α*_1_(*Q*)=*Q*′ is a *α*_1_ − transform of *Q* (see Figure 1).

**Lemma 1 .**

Let *α*_1_(*Q*)=*Q*′ be a tree derived from *Q* by *α*_1_-transform as depicted in Figure 1, then

(6) $$\begin{array}{c} F\left(\alpha_{1}\left(Q\right)\right)>F\left(Q\right). \end{array}$$

For any *p* > 1, *t* ≥ 1.

**Proof**

Observe that *d*_*Q*′_(*x*_1_)=*d*_*Q*_(*x*_1_)+*t* > *d*_*Q*_(*x*_1_) and *d*_*Q*′_(*x*_1_)+*d*_*Q*′_(*y*_1_)=*d*_*Q*_(*x*_1_)+*d*_*Q*_(*y*_1_)=*p*+*t*+2.Consider that

(7) $$\begin{array}{c} F\left(Q\prime \right)-F\left(Q\right)=\sum_{i=1}^{p}\left[\left(d_{Q\prime }{\left(x_{1,i}\right)}^{2}+d_{Q\prime }{\left(x_{1}\right)}^{2}\right)-\left(d_{Q}{\left(x_{1,i}\right)}^{2}+d_{Q}{\left(x_{1}\right)}^{2}\right)\right]\\ +\sum_{j=1}^{t}\left[\left(d_{Q\prime }{\left(y_{1,j}\right)}^{2}+d_{Q\prime }{\left(x_{1}\right)}^{2}\right)-\left(d_{Q}{\left(y_{1,j}\right)}^{2}+d_{Q}{\left(y_{1}\right)}^{2}\right)\right]\\ +\left(d_{Q\prime }{\left(x_{1}\right)}^{2}+d_{Q\prime }{\left(y_{1}\right)}^{2}\right)-\left(d_{Q}{\left(x_{1}\right)}^{2}+d_{Q}{\left(y_{1}\right)}^{2}\right)\\ =\sum_{i=1}^{p}\left[\left(d_{Q\prime }{\left(x_{1,i}\right)}^{2}+{\left(p+t+1\right)}^{2}\right)-\left(d_{Q}{\left(x_{1,i}\right)}^{2}+{\left(p+1\right)}^{2}\right)\right]\\ +\sum_{j=1}^{t}\left[\left(d_{Q\prime }{\left(y_{1,j}\right)}^{2}+{\left(p+t+1\right)}^{2}\right)-\left(d_{Q}{\left(y_{1,j}\right)}^{2}+{\left(t+1\right)}^{2}\right)\right]\\ +\left({\left(p+t+1\right)}^{2}+{\left(1\right)}^{2}\right)-\left({\left(p+1\right)}^{2}+{\left(t+1\right)}^{2}\right)\\ =3pt\left(t+p+2\right)>0. \end{array}$$

The *α*_1_ − transform decreases the degree of *y*_1_ by *t* and increases the degree of *x*_1_ by *t*, while the degrees of the nodes *x*_1,1_, *x*_1,2_,…, *x*_1,*p*_ and *y*_1,1_, *y*_1,2_,…, *y*_1,*t*_ remain unchanged.

**Lemma 2 .**

**Figure 2:**
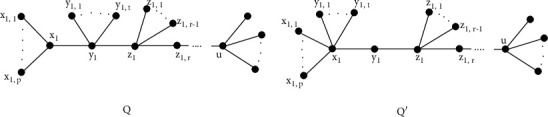
*α*_2_ − transform applied to *Q*.

Let *α*_2_(*Q*)=*Q*′ be a tree derived from *Q* as depicted in Figure 2, where *d*_*Q*_(*z*_1_, *u*) ≥ 1. Then

(8) $$\begin{array}{c} F\left(\alpha_{2}\left(Q\right)\right)>F\left(Q\right). \end{array}$$

For any *p* > 1 and *t* ≥ 1.

**Proof**

Since *d*_*Q*_(*x*_1_) < *d*_*α*_2_(*Q*)_(*x*_1_) and *d*_*α*_2_(*Q*)_(*y*_1_) < *d*_*Q*_(*y*_1_), we have

(9) $$\begin{array}{c} F\left(Q\prime \right)-F\left(Q\right)=\sum_{i=1}^{p}\left[\left(d_{Q\prime }{\left(x_{1,i}\right)}^{2}+d_{Q\prime }{\left(x_{1}\right)}^{2}\right)-\left(d_{Q}{\left(x_{1,i}\right)}^{2}+d_{Q}{\left(x_{1}\right)}^{2}\right)\right]\\ +\sum_{j=1}^{t}\left[\left(d_{Q\prime }{\left(y_{1,j}\right)}^{2}+d_{Q\prime }{\left(x_{1}\right)}^{2}\right)-\left(d_{Q}{\left(y_{1,j}\right)}^{2}+d_{Q}{\left(y_{1}\right)}^{2}\right)\right]\\ +\left(d_{Q\prime }{\left(x_{1}\right)}^{2}+d_{Q\prime }{\left(y_{1}\right)}^{2}\right)-\left(d_{Q}{\left(x_{1}\right)}^{2}+d_{Q}{\left(y_{1}\right)}^{2}\right)+\left(d_{Q\prime }{\left(y_{1}\right)}^{2}+d_{Q\prime }{\left(z_{1}\right)}^{2}\right)-\left(d_{Q}{\left(y_{1}\right)}^{2}+d_{Q}{\left(z_{1}\right)}^{2}\right)\\ =\sum_{i=1}^{p}\left[1+{\left(p+t+1\right)}^{2}-1-{\left(p+1\right)}^{2}\right]\\ +\sum_{j=1}^{t}\left[1+{\left(p+t+1\right)}^{2}-1-{\left(t+2\right)}^{2}\right]+\left[\left({\left(p+t+1\right)}^{2}+2^{2}\right)-\left({\left(p+1\right)}^{2}+{\left(t+2\right)}^{2}\right)\right]\\ +\left[\left({\left(r+1\right)}^{2}+2^{2}\right)-\left({\left(t+2\right)}^{2}+{\left(r+1\right)}^{2}\right)\right]\\ =3t\left[p\left(t+p+2\right)-\left(t+3\right)\right]>0. \end{array}$$

Hence, the result holds.

**Lemma 3 .**

**Figure 3:**
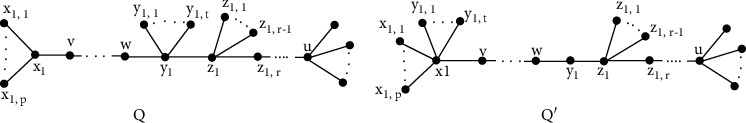
*α*_3_ − transform applied to *Q*.

Let *α*_3_(*Q*)=*Q*′ be a tree obtained from *Q* by applying *α*_3_-transform (see Figure 3), where *d*_*Q*_(*z*_1_, *u*)=*d*_*α*_3_(*Q*)_(*z*_1_, *u*) ≥ 0 and *d*_*Q*_(*x*_1_, *y*_1_)=*d*_*α*_3_(*Q*)_(*x*_1_, *y*_1_) ≥ 2. If *t* ≥ 1 and *s* > 1 then

(10) $$\begin{array}{c} F\left(\alpha_{3}\left(Q\right)\right)>F\left(Q\right). \end{array}$$

**Proof**

By definition of *F*(*Q*) we get

(11) $$\begin{array}{c} F\left(Q\prime \right)-F\left(Q\right)=\sum_{i=1}^{p}\left[\left(d_{Q\prime }{\left(x_{1,i}\right)}^{2}+d_{Q\prime }{\left(x_{1}\right)}^{2}\right)-\left(d_{Q}{\left(x_{1,i}\right)}^{2}+d_{Q}{\left(x_{1}\right)}^{2}\right)\right]\\ +\sum_{j=1}^{t}\left[\left(d_{Q\prime }{\left(y_{1,j}\right)}^{2}+d_{Q\prime }{\left(x_{1}\right)}^{2}\right)-\left(d_{Q}{\left(y_{1,j}\right)}^{2}+d_{Q}{\left(y_{1}\right)}^{2}\right)\right]\\ +\left(d_{Q\prime }{\left(x_{1}\right)}^{2}+d_{Q\prime }{\left(v\right)}^{2}\right)-\left(d_{Q}{\left(x_{1}\right)}^{2}+d_{Q}{\left(v\right)}^{2}\right)+\left(d_{Q\prime }{\left(y_{1}\right)}^{2}+d_{Q\prime }{\left(z_{1}\right)}^{2}\right)-\left(d_{Q}{\left(y_{1}\right)}^{2}+d_{Q}{\left(z_{1}\right)}^{2}\right)\\ +\left(d_{Q\prime }{\left(y_{1}\right)}^{2}+d_{Q\prime }{\left(w\right)}^{2}\right)-\left(d_{Q}{\left(y_{1}\right)}^{2}+d_{Q}{\left(w\right)}^{2}\right)=p\left[{\left(p+t+1\right)}^{2}+1-{\left(p+1\right)}^{2}\right]\\ +t\left[{\left(p+t+1\right)}^{2}+1-{\left(t+1\right)}^{2}\right]+\left[{\left(p+t+1\right)}^{2}+2^{2}\right]\\ -\left[{\left(p+1\right)}^{2}+2^{2}\right]+\left[2^{2}+2^{2}\right]-\left[2^{2}+{\left(t+2\right)}^{2}\right]+\left[2^{2}+{\left(r+1\right)}^{2}\right]-\left[{\left(t+2\right)}^{2}-{\left(r+1\right)}^{2}\right]\\ =3t\left[p\left(p+t+2\right)-\left(t+3\right)\right]>0. \end{array}$$

Hence the proof is complete.

**Lemma 4 .**

**Figure 4:**
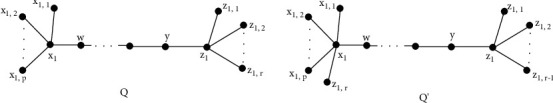
*α*_4_ − transform applied to *Q*.

*Letα*_4_(*Q*)*be a tree obtained fromQafter applyingα*_4_*-transform* (see Figure 4)*. For anyp*, *r* ≥ 0*, we have*

(12) $$\begin{array}{c} F\left(\alpha_{4}\left(Q\right)\right)>F\left(Q\right). \end{array}$$

**Proof**

If *d*_*Q*_(*x*_1_, *z*_1_) ≥ 2, then *d*_*α*_4_(*Q*)_(*x*_1_)+*d*_*α*_4_(*Q*)_(*z*_1_)=*d*_*Q*_(*x*_1_)+*d*_*Q*_(*z*_1_)=*p*+*r*+2. Now by using the definition of *F* index, we have

(13) $$\begin{array}{c} F\left(Q\prime \right)-F\left(Q\right)=\sum_{i=1}^{p}\left[\left(d_{Q\prime }{\left(x_{1,i}\right)}^{2}+d_{Q\prime }{\left(x_{1}\right)}^{2}\right)-\left(d_{Q}{\left(x_{1,i}\right)}^{2}+d_{Q}{\left(x_{1}\right)}^{2}\right)\right]\\ +\sum_{j=1}^{r-1}\left[\left(d_{Q\prime }{\left(z_{1,j}\right)}^{2}+d_{Q\prime }{\left(z_{1}\right)}^{2}\right)\right]-\sum_{j=1}^{r}\left[\left(d_{Q\prime }{\left(z_{1,j}\right)}^{2}+d_{Q\prime }{\left(z_{1}\right)}^{2}\right)\right]\\ +\left[\left(d_{Q\prime }{\left(z_{1,r}\right)}^{2}+d_{Q\prime }{\left(x_{1}\right)}^{2}\right)\right]+\left[\left(d_{Q\prime }{\left(x_{1}\right)}^{2}+d_{Q\prime }{\left(w\right)}^{2}\right)\right]-\left[\left(d_{Q}{\left(x_{1}\right)}^{2}+d_{Q}{\left(w\right)}^{2}\right)\right]\\ +\left[\left(d_{Q\prime }{\left(y\right)}^{2}+d_{Q\prime }{\left(z_{1}\right)}^{2}\right)\right]-\left[\left(d_{Q}{\left(y\right)}^{2}+d_{Q}{\left(z_{1}\right)}^{2}\right)\right]\\ =p\left[{\left(p+2\right)}^{2}+1^{2}-{\left(p+1\right)}^{2}-1\right]+\left(r-1\right)\left[r^{2}+1\right]-r\left[{\left(r+1\right)}^{2}+1\right]\\ +\left[1+{\left(p+2\right)}^{2}\right]+{\left[{\left(p+2\right)}^{2}+2\right]}^{2}-\left[{\left(p+1\right)}^{2}+2^{2}\right]+\left[2^{2}+r^{2}\right]-\left[2^{2}+{\left(r+1\right)}^{2}\right]\\ =3\left[p\left(p+3\right)-r\left(r+1\right)+2\right]>0. \end{array}$$

### 2.1. Greatest Value of *F* − Index for Trees of Given Diameter

The multistar graph denoted by *MS*(*r*_1_, *r*_2_,…, *r*_*d*−1_), where *r*_1_, *r*_*d*−1_ ≥ 1 and for 2 ≤ *j* ≤ *d* − 2, *r*_*j*_ ≥ 0, is the caterpillar involving a path *a*_1_, *a*_2_,…, *a*_*d*−1_ of length *d* − 2 having *r*_*j*_ pendant vertices that are adjacent to *a*_*j*_ for 1 ≤ *j* ≤ *d* − 1. The diameter of *MS*(*r*_1_, *r*_2_,…, *r*_*d*−1_) is equal to *d*, and can be derived by connecting the centers of *K*_1,*r*_1__, *K*_1,*r*_2__,…, *K*_1,*r*_*d*−1__ with edges. A bistar graph of order *r* denoted by *BS*(*p*, *t*), where *p*+*t*=*r* − 2, is formed by connecting the central vertices of *K*_1,*p*_ and *K*_1,*t*_ by an edge. A tree that has diameter 3 is also a bistar. For integers *r*, *t* with 2 ≤ *t* ≤ *r* − 1, *S*_*r*,*t*_ is tree derived by connecting *t* − 1 pendant vertices to the end node of the path *P*_*r*−*t*+1_, with diameter *d*(*S*_*r*,*t*_)=*r* − *t*+1.

**Theorem 1 .**

*Let*T*be a tree onr* ≥ 3*vertices and diameterd* ≥ 2*. Then the maximum value ofF*(T)*is attained for*T≅*S*_*r*,*r*−*d*+1_.

**Proof**

Applying *α*_1_-transform on the vertices that are not attached on the diametral path of T, we get that the maximum value of *F*(T) is attained in the class of multistars *MS*(*r*_1_, *r*_2_,…, *r*_*d*−1_). Now applying the transformations presented in Lemma 2–Lemma 4, it follows that the maximum value of *F*(T) is attained if and only if *r*_1_=*r* − *d*, *r*_2_=*r*_3_=⋯=0 and *r*_*d*−1_=1. Hence T≅S_*r*,*r*−*d*+1_.

**Corollary 1 .**

(i) *In the set of trees*T*onrvertices*, *we have*
  (14) $$\begin{array}{c} \max_{d\left(\text{T}\right)=i}F\left(\text{T}\right)>\max_{d\left(\text{T}\right)=j}F\left(\text{T}\right). \end{array}$$
(ii) for 2 ≤ *i* < *j* ≤ *r* − 1.
(iii) In the set of trees T of order *r* and diameter *d* with 3 ≤ *d* ≤ *r* − 2, the graphs with the greatest *F*(T) value are (in this order) as follows:
  (15) $$\begin{array}{c} \text{MS}\left(r-d,0,\ldots ,0,1\right),\text{MS}\left(r-d-1,0,\ldots ,0,2\right),\ldots ,\text{MS}\left(\left\lceil \frac{r-d+1}{2}\right\rceil ,0,\ldots ,0,\left\lceil \frac{r-d+1}{2}\right\rceil \right). \end{array}$$

**Proof**

- Let T be a tree on *r* vertices with diameter *i*. By Theorem 1 the maximum value of *F*(T) is attained for T≅*S*_*r*,*r*−*i*+1_≅*MS*(*r* − *i*, 0,…, 1). The result follows by applying many times *α*_1_-transform on *MS*(*r* − *i*, 0,…, 1).
- Applying Lemma 1–Lemma 3 to T yields the multistar MS(*p*, 0,…, 0, *q*) with *p*+*q*=*r* − *d*+1. Now using Lemma 4 to *MS*(*p*, 0,…, 0, *q*), we get the required ordering.

**Theorem 2 .**

**Figure 5:**
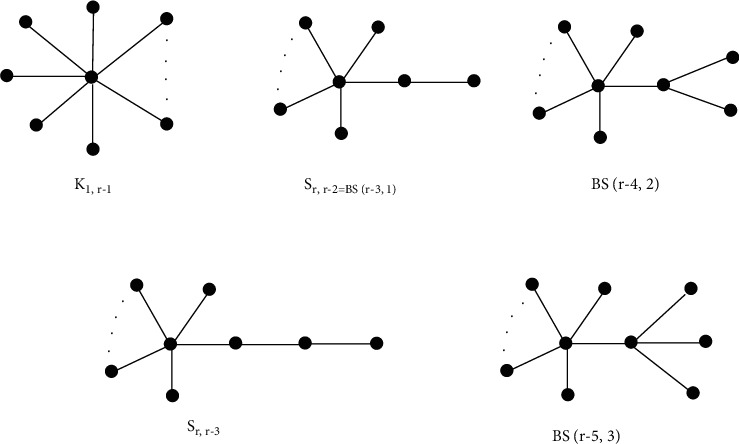
Trees T achieving greatest *F* − index.

For tree of order *r* ≥ 8, the maximum value of *F*-index is attained in the following order (see Figure 5).

(16) $$\begin{array}{c} F\left(K_{1,r-1}\right)>F\left(BS\left(r-3,1\right)\right)>F\left(BS\left(r-4,2\right)\right)>F\left(S_{r,r-3}\right)>F\left(BS\left(r-5,3\right)\right). \end{array}$$

**Proof**

Let *T* be tree of order *r* ≥ 8. By Corollary 1, the maximum value of *F*(*T*) is achieved in the set of trees of diameter 2. It follows that the trees with the maximum value of *F*(*T*) is star *K*_1,*r*−1_. The second maximum is attained for *S*_*r*,*r*−2_≅*BS*(*r* − 3,1) in the set of trees of diameter 3. The next maximum is reached by *BS*(*r* − 4,2) and *BS*(*r* − 5,3) in trees of diameter 3. Since *BS*(*r* − 3,1) can be obtained from *BS*(*r* − 4,2) by a *α*_4_ transformation, we get *F*(*K*_1,*r*−1_) > *F*(*BS*(*r* − 3,1)) > *F*(*BS*(*r* − 4,2)). In the set of trees of diameter 4, the maximum value is attained by *S*_*r*,*r*−3_. To get the fourth maximum value we compare *F*(*BS*(*r* − 5,3)) with *F*(*S*_*r*,*r*−3_). We have

(17) $$\begin{array}{c} F\left(BS\left(r-5,3\right)\right)-F\left(S_{r,r-3}\right)=\left[\left(r-5\right)\left\{1+{\left(r-4\right)}^{2}\right\}+\left\{{\left(r-4\right)}^{2}+16\right\}+3\left(17\right)\right]\\ -\left[\left(r-4\right)\left\{1+{\left(r-3\right)}^{2}\right\}+\left\{{\left(r-3\right)}^{2}+4\right\}+8+5\right]=-3r^{2}+21r+12<0. \end{array}$$

Hence *F*(*BS*(*r* − 5,3)) < *F*(*S*_*r*,*r*−3_) for every *r* ≥ 8. Also,

(18) $$\begin{array}{c} F\left(BS\left(r-5,3\right)\right)-F\left(MS\left(r-5,0,2\right)\right)=\left[\left(r-5\right)\left\{1+{\left(r-4\right)}^{2}\right\}+\left\{{\left(r-4\right)}^{2}+16\right\}+3\left(17\right)\right]\\ -\left[\left(r-5\right)\left\{{\left(r-4\right)}^{2}+1\right\}+\left\{{\left(r-4\right)}^{2}+4\right\}+13+20\right]=30>0. \end{array}$$

This shows that the second maximum value of *F*-index is achieved by *MS*(*r* − 5,0,2) after *S*_*r*,*r*−3_ in the set of trees of diameter 4. Using a *α*_1_-transform, it is easy to see that *MS*(*r* − 5,0,0,1) reaches the maximum value in the set of trees of diameter 5 and *F*(*MS*(*r* − 5,0,0,1)) < *F*(*MS*(*r* − 5,0,2)), which completes the proof.

**Example 1 .**

Let T be a tree on 10 vertices, then

(19) $$\begin{array}{c} F\left(K_{1,10-1}\right)=\sum_{uv\in E\left(Q_{1,10-1}\right)}\left(d^{2}\left(u\right)+d^{2}\left(v\right)\right)=9\left(1^{2}+9^{2}\right)=738,\\ F\left(BS\left(10-3,1\right)\right)=\sum_{uv\in E\left(BS\left(10-3,1\right)\right)}\left(d^{2}\left(u\right)+d^{2}\left(v\right)\right)=\left[1^{2}+2^{2}\right]+\left[2^{2}+8^{2}\right]+7\left[1^{2}+8^{2}\right]=528,\\ F\left(BS\left(10-4,2\right)\right)=\sum_{uv\in E\left(BS\left(10-4,2\right)\right)}\left(d^{2}\left(u\right)+d^{2}\left(v\right)\right)=2\left[1^{2}+3^{2}\right]+\left[3^{2}+7^{2}\right]+6\left[1^{2}+7^{2}\right]=378,\\ F\left(S_{10,10-3}\right)=\sum_{uv\in E\left(S_{10,10-3}\right)}\left(d^{2}\left(u\right)+d^{2}\left(v\right)\right)=\left[1^{2}+2^{2}\right]+\left[2^{2}+2^{2}\right]+\left[2^{2}+7^{2}\right]+6\left[1^{2}+7^{2}\right]=366,\\ F\left(BS\left(10-5,3\right)\right)=\sum_{uv\in E\left(10-5,3\right)}\left(d^{2}\left(u\right)+d^{2}\left(v\right)\right)=3\left[1^{2}+4^{2}\right]+\left[4^{2}+6^{2}\right]+5\left[1^{2}+6^{2}\right]=288. \end{array}$$

This shows that

(20) $$\begin{array}{c} F\left(K_{1,10-1}\right)>F\left(BS\left(10-3,1\right)\right)>F\left(BS\left(10-4,2\right)\right)>F\left(S_{10,10-3}\right)\\ >F\left(BS\left(10-5,3\right)\right). \end{array}$$

**Theorem 3 .**

*In the set of trees*T*of orderr* ≥ 5*withtpendant vertices, where*3 ≤ *t* ≤ *r* − 2*, we have*

(21) $$\begin{array}{c} F\left(T\right)\leq t^{3}-7t+8r-8. \end{array}$$

The above equality holds if and only if *T*=*S*_*r*,*t*_.

**Proof**

First we prove that if *x* is a pendant vertex adjacent to *y*, then

(22) $$\begin{array}{c} F\left(T\right)-F\left(T-x\right)\leq 3t^{2}-3t+2. \end{array}$$

With equality holds if and only if *T*=*S*_*r*,*t*_ and *d*(*y*)=*t*. Since 3 ≤ *t* ≤ *r* − 2, it follows that there exists a vertex *z*_0_ ∈ *N*(*y*)/{*x*}, such that *d*_*z*_0__ ≥ 2. Otherwise *T* is a star having central vertex *y*. We obtain

(23) $$\begin{array}{c} F\left(T\right)-F\left(T-x\right)=\left(d{\left(y\right)}^{2}+1\right)-\sum_{z\in N\left(y\right)/\left\{x\right\}}\left[d{\left(z\right)}^{2}+{\left(d\left(y\right)-1\right)}^{2}-\left(d{\left(z\right)}^{2}+d{\left(y\right)}^{2}\right)\right]. \end{array}$$

Since, *d*(*z*_0_) ≥ 2 and for the remaining *d*(*y*) − 2 nodes *z* ∈ *N*(*y*)/{*x*, *z*_0_} , where *d*(*z*) ≥ 1, we have

(24) $$\begin{array}{c} F\left(T\right)-F\left(T-x\right)=\left(3d{\left(y\right)}^{2}-3d\left(y\right)+2\right). \end{array}$$

We also get *d*(*y*) ≤ *t* because *T* − *y* includes of *d*(*y*) trees. Now 2 ≤ *d*(*y*) ≤ *t* gives

(25) $$\begin{array}{c} F\left(T\right)-F\left(T-x\right)\leq \left(3t^{2}-3t+2\right), \end{array}$$

with equality holds if *d*(*y*)=*t*, the adjacent vertex of *y* has degree 2 and the remaining vertices are of degree 1. Hence *T*=*S*_*r*,*t*_ and *x* is adjacent to one of the vertex of *S*_*r*,*t*_ of degree *t*.

Now we use induction to prove the required result. If *r*=5, then *t*=3 and we have a bistar *BS*(2,1) (see Figure 5), the only tree of order 5 having 3 pendant vertices. Let *r* ≥ 6 and suppose the result is true for all trees of order *r* − 1 and *t* pendant vertices, where 3 ≤ *t* ≤ *r* − 3. Let *x* be a pendant vertex adjacent to *y*, then we have two cases: (i) *y* has degree 2 and (ii) *y* is of degree at most 3.

(i) The only vertex *z* adjacent to *y* has degree *d*(*z*) ≥ 2. Then
  (26) $$\begin{array}{c} F\left(T\right)-F\left(T-x\right)=\left(d{\left(z\right)}^{2}+4\right)+5-\left(d{\left(z\right)}^{2}+1\right)=8. \end{array}$$
(ii) In this case the graph *T* − *x* has *t* pendant vertices. Applying induction, for *t* ≤ *r* − 3, we get *F*(*T* − *x*) ≤ *F*(*S*_*r*−1,*t*_), with equality holds for T − *x*=S_*r*−1,*t*_. It follows that
  (27) $$\begin{array}{c} F\left(T\right)=F\left(T-x\right)+8\leq F\left(S_{r-1,t}\right)+8=F\left(S_{r,t}\right), \end{array}$$
(iii) with equality holds for *T* − *x*=*S*_*r*−1,*t*_. Therefore, we have *T*=*S*_*r*,*t*_. If *t*=*r* − 2, then *T* − *x* is a star with one vertex of degree 1. Hence, *T*=*S*_*r*,*r*−2_=*S*_*r*,*t*_.
(iv) Let *T* − *x* is of order *r* − 1 with *st* − 1 pendant vertices. If *d*(*y*) ≥ 3, then by using induction on *T* − *x*, we get
  (28) $$\begin{array}{c} F\left(T\right)\leq F\left(T-x\right)+3t^{2}-3t+2\leq F\left(S_{r-1,t-1}\right)+3t^{2}-3t+2=F\left(S_{r,t}\right), \end{array}$$

with equality holds for *T* − *x*=*S*_*r*−1,*t*−1_ and *d*(*y*)=*t*. Hence *T*=*S*_*r*,*t*_.

## 3. Conclusion

In this paper, our main focus is to obtain the greatest value of *F-index* for trees of given order, diameter, and pendent vertices. Also, we determine the ordering of corresponding extremal trees for *F-index*. We make use of some graph transformations to determine the greatest value of *F-index* for trees of given order, diameter, and pendent vertices. These transformations involves contraction and swapping of pendant edges from one vertex to other resulting in increase in the value of the forgotten index. Using these transformations continuously on a graph lead us to the desired extremal graph with respect to the forgotten index. On the way of obtaining extremal graphs, we also obtained some other members having the second, third, fourth, and fifth maximum value of the forgotten index.

## Data Availability

No data were used to support this study.
